# Mesenchymal stromal cell-laden hydrogels in tissue regeneration: insights from preclinical and clinical research

**DOI:** 10.3389/fphar.2025.1670649

**Published:** 2025-09-12

**Authors:** Silvia Barbon, Senthilkumar Rajendran, Antara Banerjee, Pier Paolo Parnigotto, Raffaele De Caro, Veronica Macchi, Andrea Porzionato

**Affiliations:** ^1^ Department of Neuroscience, Section of Human Anatomy, University of Padua, Padua, Italy; ^2^ Foundation for Biology and Regenerative Medicine, Tissue Engineering and Signaling - T.E.S. Onlus, Padova, Italy; ^3^ Faculty of Allied Health Sciences, Chettinad Academy of Research and Education (CARE), Chettinad Hospital and Research Institute (CHRI), Chennai, India

**Keywords:** mesenchymal stromal cells, hydrogels, tissue regeneration, preclinical research, clinical trials

## Abstract

Hydrogel-based delivery systems have emerged as a promising strategy to enhance the therapeutic efficacy of mesenchymal stromal cells (MSCs) in regenerative medicine. These biomimetic platforms provide a three-dimensional microenvironment that recapitulates key features of native extracellular matrix, supporting MSC viability, retention, and function upon transplantation. Beyond acting as passive carriers, hydrogels can be engineered with tunable biochemical and mechanical properties to modulate MSC behavior, including their differentiation potential, immunomodulatory activity, and paracrine signaling. Recent advances include the development of “smart” hydrogels responsive to physiological stimuli, enabling controlled release of encapsulated cells or bioactive molecules in response to local cues. Preclinical studies have demonstrated enhanced tissue repair in diverse pathological contexts, including musculoskeletal, cardiovascular, gastrointestinal, dermal, and neural injuries. Importantly, translation to clinical settings is being facilitated by the use of xeno-free, good manufacturing practices (GMP)-compliant components such as platelet derivatives and synthetic polymers. Selected early-phase clinical trials support the feasibility, safety, and therapeutic potential of MSC-laden hydrogels, although further studies are required to optimize delivery parameters and regulatory compliance. This review summarizes current progress in hydrogel-MSC systems across application areas, emphasizing design principles, preclinical outcomes, and translational challenges, with the aim of guiding future developments in stem cell-based tissue regeneration.

## 1 Introduction

Tissue regeneration remains a significant challenge in modern medicine, particularly for the restoration of structure and function in tissues damaged by trauma, chronic disease, or degenerative conditions related to aging. The intrinsic complexity of tissue architecture and the inflammatory and fibrotic responses following injury often hinder spontaneous healing and limit the efficacy of conventional therapeutic strategies. In this context, regenerative medicine research aims to harness the therapeutic potential of stem cells to develop more effective, biologically integrated strategies for tissue repair and functional restoration (Hoang et al., 2022; Hussen et al., 2024).

Among the various cell types explored for regenerative purposes, mesenchymal stromal cells (MSCs) have emerged as one of the most promising candidates due to their ease of isolation, multipotent differentiation capacity, secretion of trophic factors, and immunomodulatory properties (Fan X. L. et al., 2020; Barbon et al., 2022; Barbon et al., 2021). These cells can contribute to tissue repair through both direct differentiation into target cell lineages and through paracrine mechanisms that modulate inflammation, promote angiogenesis, and recruit endogenous progenitor cells (Kaviarasan et al., 2024; Xue et al., 2024; Barbon et al., 2023).

However, the effective delivery and retention of MSCs at the injury site remain critical hurdles. Rapid cell death, washout due to mechanical forces, and lack of a supportive microenvironment compromise their regenerative potential (Bagno et al., 2022; Takayama et al., 2023). In particular, recent analyses have highlighted that cell-based interventions alone frequently fail to achieve long-term and uncomplicated wound healing across different tissue contexts. This limitation has been attributed to the transient survival of transplanted MSCs and the short-lived duration of their anti-inflammatory secretory activity, which may be insufficient to sustain the prolonged phases of tissue repair and remodeling (Zhao et al., 2021).

To overcome these barriers, biomaterial-based strategies have been developed to provide scaffolding for cell delivery, with hydrogels standing out as an optimal solution. Hydrogels are water-swollen, crosslinked polymer networks that closely mimic the physical and biochemical properties of the native extracellular matrix (ECM). Their biocompatibility, tunable mechanical strength, and ability to encapsulate and release cells or bioactive molecules make them ideal vehicles for supporting MSC survival and function (Seliktar, 2012).

The combination of MSCs and hydrogels has thus gained considerable attention in regenerative medicine, offering a synergistic approach to enhance tissue regeneration. Recent preclinical and clinical studies have explored the potential of MSC-laden hydrogels in various applications, including cartilage repair, wound healing, and myocardial regeneration (Gnecchi et al., 2016; Liang et al., 2019). Despite promising results, challenges such as optimal hydrogel composition, cell viability, and long-term therapeutic efficacy remain to be addressed.

This literature review examines the current preclinical and clinical research on MSC-laden hydrogels, evaluating their therapeutic outcomes, limitations, and future directions in tissue regeneration. By synthesizing findings from key studies, this review aims to provide a comprehensive understanding of the translational potential of MSC-hydrogel systems in regenerative medicine. Given the extensive body of research on hydrogel-assisted MSC delivery, this work does not aim to provide an exhaustive systematic review. Instead, a focused narrative approach was adopted, selecting representative preclinical and clinical studies that best illustrate the translational potential of MSC-laden hydrogels. Specifically, we prioritized *in vivo* investigations in clinically relevant animal models, along with published and ongoing clinical trials in humans. In vitro-only studies, early proof-of-principle experiments, and review articles were excluded from the scope. This strategy allows us to emphasize the therapeutic promise, current limitations, and future perspectives of MSC-hydrogel constructs within a clinically oriented framework.

## 2 Hydrogel properties enhancing MSC function

Hydrogels play a pivotal role in optimizing the therapeutic efficacy of MSCs for tissue regeneration by providing a biomimetic three-dimensional (3D) microenvironment that closely resembles the native ECM. This supportive niche not only facilitates MSC viability and engraftment but also modulates their paracrine and immunomodulatory functions. The mechanical and biochemical properties of hydrogels significantly influence MSC behavior, including cell survival, proliferation, migration, and lineage-specific differentiation, thereby directly impacting the regenerative process (Garcia-Aponte et al., 2025).

Hydrogels with tunable stiffness, porosity, and degradation kinetics can be engineered to mimic the mechanical properties of specific target tissues (Barbon et al., 2020; Todros et al., 2022a; Todros et al., 2022b). For example, softer hydrogels with elastic moduli in the range of 1–10 kPa have been shown to promote adipogenic or neurogenic differentiation, whereas stiffer matrices ranging from 25 to 40 kPa tend to favor osteogenic commitment (Engler et al., 2006). This mechanosensitivity underscores the importance of substrate stiffness in guiding stem cell fate decisions (Stocco et al., 2019a). Furthermore, pore architecture affects nutrient diffusion, waste elimination, and cell migration, all of which are essential for maintaining a viable and functionally active MSC population *in situ* (Zhou et al., 2023). Complementing these internal features, hydrogel surface geometry - including features such as roughness, curvature, and micro- or nano-topography - plays a critical role in modulating MSC adhesion, proliferation, and lineage commitment (Xiao et al., 2023; Stocco et al., 2021; Stocco et al., 2024; Di Liddo et al., 2016). These topographical cues can influence cytoskeletal organization and mechanotransduction pathways, thereby directing stem cell differentiation and enhancing tissue-specific integration.

The incorporation of bioactive molecules, such as ECM-derived peptides [e.g., arginine–glycine–aspartic acid (RGD), laminin], growth factors [e.g., vascular endothelial growth factor (VEGF), fibroblast growth factor-2 (FGF-2), bone morphogenetic protein-2 (BMP-2)], and glycosaminoglycans (e.g., hyaluronic acid or chondroitin sulfate), further augments MSC functionality by facilitating cell adhesion, activating integrin-mediated signaling pathways, and enhancing the secretion of regenerative cytokines (Xiao et al., 2023; Porzionato et al., 2019; Di Liddo et al., 2014). These biochemical cues can be spatially and temporally regulated through hydrogel composition or surface modification, allowing more precise control of the cellular microenvironment.

Injectable hydrogels, including those based on natural polymers such as alginate, collagen, gelatin, or hyaluronic acid, enable minimally invasive administration, *in situ* gelation, and conformation to irregular defect geometries. This ensures precise MSC localization, retention, and protection within injured tissues (Burdick and Murphy, 2012). Synthetic variants such as polyethylene glycol (PEG) and polyvinyl alcohol (PVA) offer improved mechanical tunability and reproducibility, though often at the expense of bioactivity. Composite hydrogels combining natural and synthetic components aim to leverage the advantages of both material classes.

In addition to traditional natural and synthetic polymers, hydrogels derived from decellularized ECM have gained increasing attention as MSC carriers. These biomaterials closely mimic the native biochemical composition and architecture of tissues, thereby providing a bioactive microenvironment that promotes cell adhesion, survival, and lineage-specific differentiation. However, the intrinsic mechanical weakness and batch-to-batch variability of pure ECM hydrogels may limit their translational application. To address this, bio-hybrid systems combining ECM components with synthetic polymers have been developed, combining the bioactivity of ECM with the tunable mechanical and physicochemical properties of synthetic materials (Stocco et al., 2014; Stocco et al., 2016; Grandi et al., 2018; Stocco et al., 2022). Such composite scaffolds sowed to enhance MSC retention and regenerative capacity across different tissues, including cartilage, intestinal segments, and osteochondral units. *In vitro* and preclinical evidence further highlighted that ECM-derived hydrogels not only reproduce key integrin-binding and growth factor-retaining motifs, but also demonstrate promising preclinical outcomes in cartilage, bone, cardiac, and cutaneous repair. Based on that, hybrid ECM-synthetic systems emerge as promising next-generation carriers for MSC transport, capable of coupling structural stability with biochemical functionality to better meet the complex demands of tissue regeneration (Soltanmohammadi et al., 2025).

**FIGURE 1:**
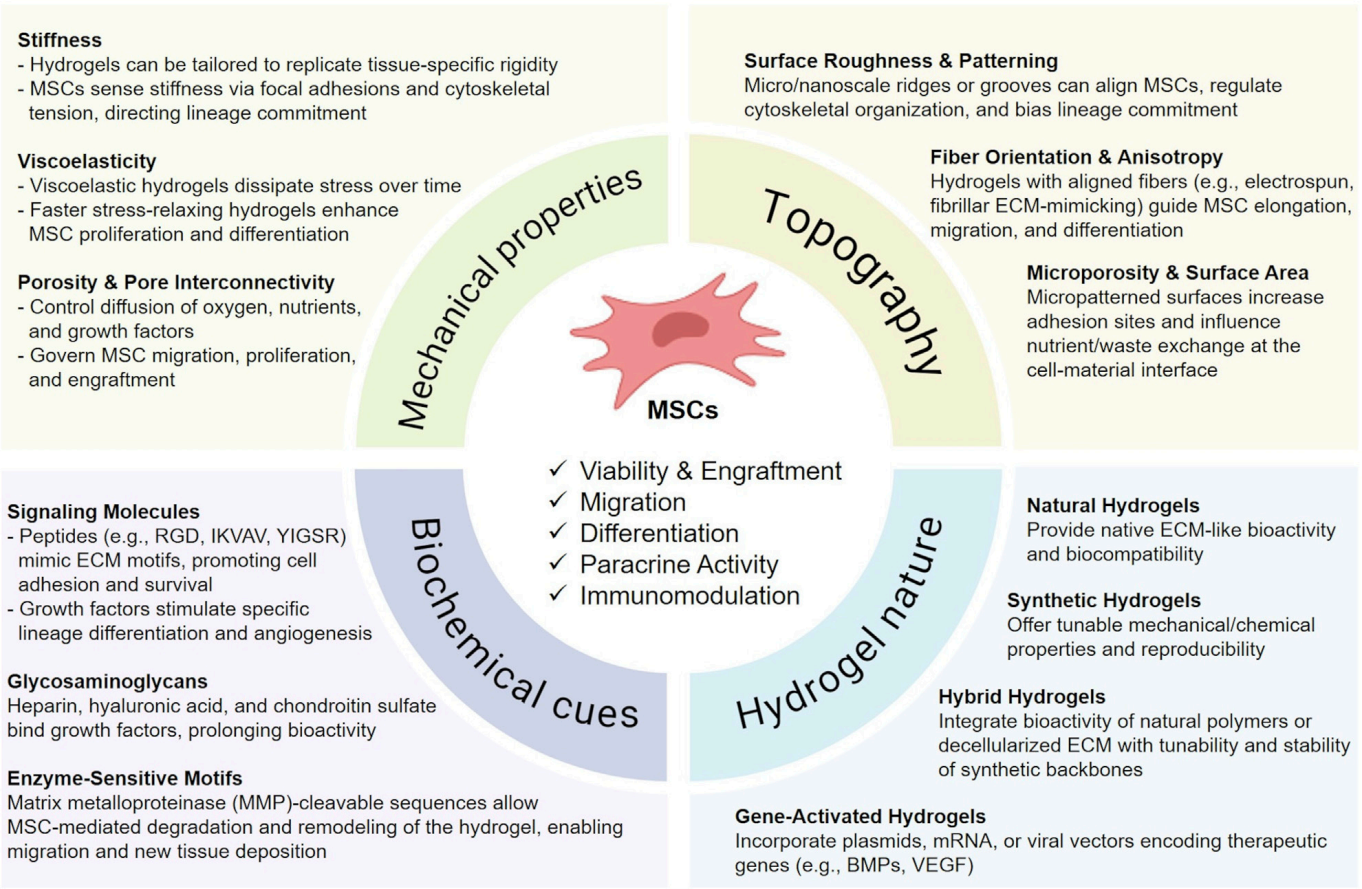
Key hydrogel design parameters influencing MSC therapeutic activity. Schematic overview of hydrogel features that regulate mesenchymal stromal cell (MSC) function and regenerative potential. Mechanical properties such as stiffness, viscoelasticity, and porosity govern MSC survival, migration, and lineage commitment. Topographical cues, including surface roughness, fiber alignment, and microporosity, modulate cell adhesion, elongation, and differentiation. Biochemical signals, provided by peptides, growth factors, glycosaminoglycans, and enzyme-sensitive motifs, enhance MSC survival, angiogenesis, and remodeling capacity. The intrinsic nature of the hydrogel further impacts therapeutic outcomes: natural hydrogels ensure ECM-like bioactivity, synthetic hydrogels provide reproducibility and tunability, hybrid systems combine both advantages, and gene-activated hydrogels enable controlled release of therapeutic genes. Collectively, these design parameters influence MSC viability, engraftment, paracrine activity, and immunomodulation in regenerative applications. This figure was created using BioRender.com.

Parallel to these bio-hybrid strategies, advanced designs such as stimuli-responsive or dynamic hydrogels, incorporate environmental triggers (e.g., pH, temperature, enzymatic activity) to enable the controlled release of encapsulated cells or bioactive factors. These “smart” hydrogels can prolong therapeutic action, support tissue remodeling, and potentially provide on-demand modulation of MSC activity (Lu et al., 2024) (Figure 1).

A promising strategy to further refine hydrogel bioactivity involves engineering matrices with controlled surface or volumetric charge. (e.g., heparinization to confer anionic binding domains or incorporation of cationic moieties) to non-covalently sequester nucleic acids (microRNA, mRNA, plasmid DNA). Such gene-activated hydrogels can prolong local factor residence, protect labile cargos from degradation, and enable cell-responsive release, thereby extending and amplifying MSC paracrine activity *in vivo*. Preliminary studies further indicate that charge density and spatial distribution critically regulate payload loading/release kinetics and downstream MSC immunomodulation and differentiation (Krasilnikova et al., 2023; Liu et al., 2024).

Despite their potential, hydrogel-based strategies still have to face significant challenges. A key consideration is aligning hydrogel degradation rates with the timeline of native tissue healing to avoid premature scaffold loss or prolonged presence that could hinder integration. Batch-to-batch variability in natural polymers and potential immunogenicity also pose hurdles to clinical translation. Meanwhile, synthetic hydrogels often lack intrinsic bioactivity, requiring functionalization strategies to enhance their biological performance.

Future directions should prioritize the development of clinically scalable, off-the-shelf hydrogel-MSC formulations with optimized degradation kinetics, mechanical resilience, and biofunctionality. Strategies incorporating extracellular vesicles (EVs) or gene editing tools may further enhance therapeutic outcomes by augmenting MSC paracrine signaling or resistance to hostile injury microenvironments.

## 3 Paracrine signaling and immunomodulation

**FIGURE 2:**
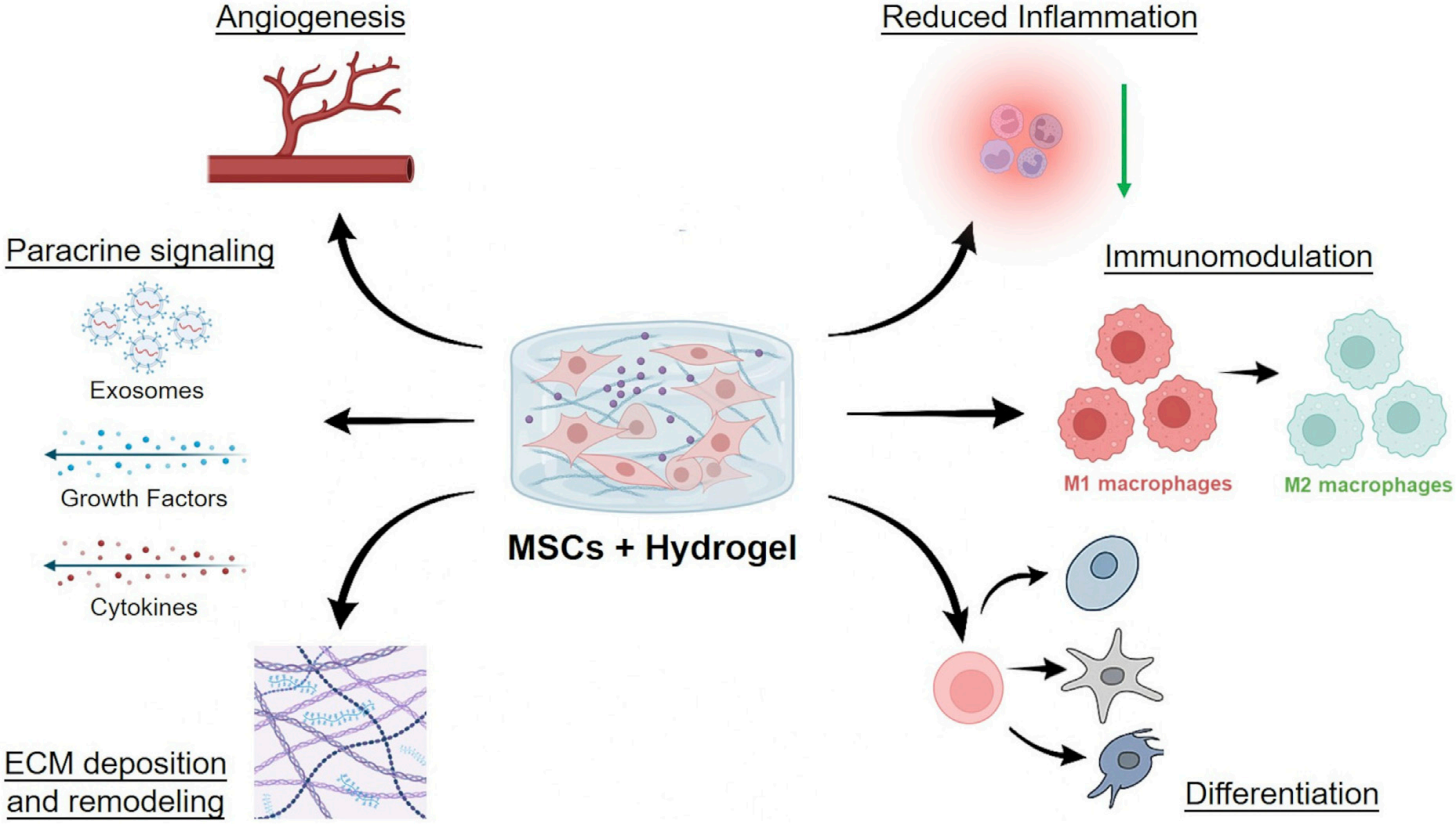
Mechanisms of MSC delivery and action within hydrogels. Schematic representation of how mesenchymal stromal cells (MSCs) act when encapsulated in hydrogel scaffolds. Hydrogels provide a protective scaffold that enhances MSC survival and retention, while enabling multiple therapeutic mechanisms. Paracrine signaling through cytokines, exosomes, and growth factors promotes angiogenesis and tissue repair. Immunomodulation drives macrophage polarization from pro-inflammatory M1 to anti-inflammatory M2 phenotypes, resulting in reduced inflammation. Differentiation into lineage-specific cells (e.g., chondrocytes, osteoblasts, neurons) and matrix deposition and remodeling contribute to structural regeneration. Collectively, these processes support vascularization, immune regulation, and tissue repair in regenerative medicine applications. This figure was created using BioRender.com.

Mesenchymal stromal cell (MSC)-laden hydrogels exert their regenerative effects primarily through paracrine signaling and immunomodulation rather than direct differentiation. MSCs encapsulated in hydrogels secrete a diverse array of bioactive factors, including growth factors [e.g., VEGF, hepatocyte growth factor (HGF), transforming growth factor beta (TGF-β)], cytokines [e.g., interleukin-10 (IL-10), tumor necrosis factor-stimulated gene-6 (TSG-6)], and extracellular vesicles (exosomes), which collectively promote tissue repair by stimulating angiogenesis, reducing fibrosis, and modulating immune responses (Kuppa et al., 2022). The hydrogel matrix serves as a protective niche that prolongs MSC survival and sustains the release of these therapeutic factors, enhancing their local bioavailability compared to free MSC (Caplan and Correa, 2011) (Figure 2).

A key advantage of MSC-laden hydrogels is their ability to polarize macrophages from a pro-inflammatory (M1) to an anti-inflammatory (M2) phenotype, which is critical for resolving chronic inflammation in conditions like diabetic wounds or myocardial infarction (Saldanha-Araujo et al., 2020). Hydrogels can be further engineered to amplify these effects by incorporating immunomodulatory agents (e.g., IL-4, interferon gamma (IFN-γ)) or ECM components that synergize with MSC secretions (Xie et al., 2020).

Despite these benefits, challenges include variability in MSC secretomes due to donor differences and culture conditions, as well as the short-lived activity of some paracrine factors. Future strategies may involve genetic modification of MSCs to overexpress specific factors or the use of synthetic hydrogels with controlled release kinetics to optimize immunomodulatory outcomes. Importantly, in the context of wound healing stimulation, these paracrine effects may decrease before tissue repair is stabilized, resulting in incomplete, not functional regeneration. Hydrogel systems are being explored specifically to prolong factor release, modulate local immunity, and synchronize MSC activity with the protracted timeline required for durable and uncomplicated wound closure.

## 4 Hydrogel-based MSC therapies: preclinical studies

Preclinical studies represent a fundamental step in validating the therapeutic and translational potential of MSC-hydrogel systems across a wide range of disease models. Animal experiments provide essential proof-of-concept data and mechanistic insights into how biomaterial-assisted MSC therapy can overcome limitations such as poor cell survival, retention, and integration. Importantly, the outcomes of these studies depend strongly on the physiological niche in which the therapy is applied. In ischemic tissues, hydrogels protect MSCs from hypoxia and modulate macrophage-driven fibrosis; in neuroinhibitory niches, they attenuate glial scarring and reprogram microglia; in immune-active tissues such as skin and gut, they regulate inflammatory cascades; in mechanically demanding tissues like cartilage and bone, they provide physical support and engage osteo-immune cells; and in sensitive tissues, scaffold selection must minimize excessive immune activation. To reflect this, the following subsections review representative preclinical applications of hydrogel-assisted MSC delivery according to the major biological challenges that influence regenerative success. This integrated perspective highlights both the similarities and the context-specific differences across models, underscoring how hydrogel design can be strategically adapted to distinct tissue environments and host responses, and illustrating the versatility of MSC–hydrogel constructs across a spectrum of regenerative contexts (Table 1).

**TABLE 1:** Overview of MSC laden-hydrogel preclinical research.

First author, year	Clinical target	Type of MSCs	Type of hydrogel	Animal model, tissue damage	Main *in vivo* outcomes
Wang et al. (2010)	Cartilage repair	BM-MSCs	PLGA sponge filled with fibrin gel + TMC/pDNA-TGF-β1 complexes	Rabbit, full-thickness femoral trochlear cartilage defect	- Hyaline-like cartilage formation with abundant type II collagen and GAGs - Improved surface smoothness and better integration - Evident subchondral bone remodeling
Chen et al. (2011)	Osteochondral regeneration	BM-MSCs	Bilayer gene-activated scaffold: a) plasmid TGF-β1-activated chitosan-gelatin as chondrogenic layer b) plasmid BMP-2-activated hydroxyapatite/chitosan-gelatin as osteogenic layer	Rabbit, knee osteochondral defect	- Simultaneous regeneration of cartilage and subchondral bone - Superior ICRS/Wakitani-type histology scores - Optimal cartilage–bone integration
Levit et al. (2013)	Myocardial infarction	BM-MSCs	Alginate-based encapsulating hydrogel	Rat LAD ligation	- Improved cell retention, cardiac function, and neovascularization
Li et al. (2013)	Cartilage repair	BM-MSCs	PLGA scaffold + fibrin gel + PEO-b-PLL/TGF-β1 plasmid DNA complexes	Rabbit, osteochondral defect	- Enhanced chondrogenesis - Higher histological repair scores - Improved defect fill and integration - More homogeneous hyaline-like tissue
Seol et al. (2013)	Cartilage repair/pain control	BM-MSCs	Reverse-thermal responsive Poloxamer hydrogel	Rabbit Articular cartilage lesion	- High hydrogel biocompatibility - Hyaline-like cartilage regeneration with smooth surface morphology - Good tissue integration, localized cell retention - No adverse immune reactions
Kopesky et al. (2014)	Cartilage repair	BM-MSCs	Self-assembling peptide hydrogel (TGF-β1 delivery)	Horse, articular cartilage defects	- Induced chondrogenesis - High glycosaminoglycan and collagen II deposition
Ha et al. (2015)	Cartilage repair	UCB-MSCs	Hyaluronic acid hydrogel	Minipig, full-thickness cartilage defect	- Hyaline-like cartilage regeneration - Good integration with native tissue
Bartlett et al. (2016)	Vocal fold repair	BM- MSCs	Hyaluronic acid hydrogel	Dog, vocal fold scarring model	- Decreased fibrosis - Partial restoration of vocal fold elasticity
Borg et al. (2016)	Pancreatic islet support	BM-MSCs	Macroporous PEG cryogel (heparin-functionalized)	Mouse, diabetic islet transplant model	- Improved islet viability - Immune modulation - Enhanced insulin secretion
Kolakshyapati et al. (2017)	Skin/sweat glands regeneration	BM-MSCs	Collagen-chitosan porous scaffold + Lipofectamine 2000/pDNA-EGF complexes	Mouse, full-thickness skin wound	- Formation of sweat-gland–like structures in regenerated skin - Upregulation of sweat-gland markers - Improved re-epithelialization and dermal remodeling
Lin et al. (2017)	Bone regeneration	BM-MSCs	Projection stereolithography-fabricated gene-activated matrix (BMP-2 pDNA embedded in photo-crosslinked hydrogel)	Mouse, implantation into the intramuscular compartment of upper long bone	- Robust ectopic bone formation - Mineralize bone and vascularization detected by histological studies
Wu et al. (2017)	Myocardial infarction	MSCs	Small-molecule self-assembling hydrogel	Rat LCA ligation	- Improved LVEF and fractional shortening - Reduced scar area and increased capillary density - Higher cell retention within infarct border zone
Papa et al. (2018)	Spinal cord injury	BM-MSCs	PEG hydrogel releasing CCL2	Mouse, contusive SCI	- Preserved spinal cord architecture - Reduced inflammation - Improved locomotor recovery
Loozen et al. (2019)	Bone regeneration	MSCs	Alginate-ceramic scaffold with BMP-2 gene delivery	Rat, spinous process defect at the lumbosacral junction	- Robust bone regeneration - Substantial defect fill and restored bone volume at the micro-CT analysis - Histological evidence of well-organized, mineralized bone bridging the defect
Moussa et al. (2019)	Radiation-induced colonic damage	BM-MSCs	Heparan sulfate mimetic alginate-based hydrogel	Rat, localized colonic irradiation	- Reduced epithelial injury - Reduced colonic inflammation - Improved epithelial regeneration and restoration of mucosal architecture
Black et al. (2020)	Bone regeneration	Stro-4+ enriched MSCs	Bovine bone ECM hydrogel with PCL scaffold	Rat, femoral defect	- Enhanced mineralization, bone volume, and osteointegration
Sun et al. (2020)	Bone regeneration	BM-MSCs	Methacrylated gelatin-based hydrogel + rAAV encoding BMP2	Mouse, calvarial bone defect	- Accelerated bone bridging with higher bone volume and cortical continuity - Robust osteogenesis and vascularization - Mature trabecular bone tissue formation
Vivas et al. (2020)	Bone regeneration	BM-MSCs	Collagen-based GMP-grade hydrogel	Mouse, calvarial bone defect	- Early bone formation and vascularization - Clinically compliant MSC-hydrogel formulation
Zhang et al. (2021)	IVD degeneration	BM-MSCs	Fibrin-genipin hydrogel	Goat, moderate disc degeneration model	- Restored disc height and proteoglycan content - Reduced inflammatory cytokines
Lee et al. (2022)	Atopic dermatitis	MSCs	Thiolated hyaluronic acid hydrogel	Mouse DNCB-induced dermatitis	- Reduced epidermal thickness - Lowered cytokine expression - Improved skin barrier integrity
Canceill et al. (2023)	MSC delivery platform as ATMP	BM-MSCs	Platelet lysate-based fibrin hydrogel	Mouse, subcutaneous implantation	- No adverse immune reactions - Preserved MSC viability and differentiation potential
Hu et al. (2024)	Osteochondral regeneration	BM-MSCs	Nanozyme-functionalized bilayer hydrogel (oxidized dextran/gelatin + bioactive layer)	Rat, full-thickness osteochondral defect	- Improved cartilage repair - Subchondral bone formation - Reduced synovial inflammation
Li et al. (2024)	Spinal fusion	MSCs	Gelatin-methacryloyl composite hydrogel with BMP-2	Rat, posterolateral spinal fusion model	- Enhanced bone formation - Increased fusion rates - Osteoinduction
Huang et al. (2025)	Diabetic wound healing	Perinatal MSCs	Injectable PEG-based hydrogel	Mouse, streptozotocin-induced diabetic wounds	- Faster wound closure - Increased angiogenesis - Re-epithelialization via PI3K/AKT signaling
Sun et al. (2025)	Spinal cord injury	BM-MSCs	BMP7-loaded gelatin methacryloyl hydrogel	Rat T10 hemisection SCI	- Increased neuronal marker expression - Enhanced axon regeneration - Motor function recovery
Tavajjohi et al. (2025)	Myocardial infarction	Wharton’s Jelly MSCs	Wharton’s Jelly ECM-derived hydrogel	Rat LAD ligation	- Reduced infarct size - Enhanced angiogenesis - Improved ejection fraction - Decreased fibrosis

ATMP, advanced therapy medicinal product; BM-MSCs, Bone Marrow Mesenchymal Stromal Cells; BMP; Bone Morphogenetic Protein; DNCB, 2,4-dinitrochlorobenzene; ECM, Extracellular Matrix; EGF, Epidermal Growth Factor; GAGs, Glycosaminoglycans; ICRS, International Cartilage Repair Society; IVD, Intervertebral Disc; LAD, Left Anterior Descending Artery; LCA, Left Coronary Atery; LVEF, Left Ventricular Ejection Fraction; MSCs, Mesenchymal Stromal Cells; PCL, Polycaprolactone; pDNA, plasmid DNA; PEG, Polyethylene Glycol; PEO-b-PLL, poly (ethylene oxide)-b-poly (L-lysine); PI3K/AKT, phosphoinositide 3-kinase/protein kinase B; PLGA, Poly (Lactide-co-Glycolide); rAAV, recombinant adeno-associated viral vector; SCI, Spinal Cord Injury; TGF, Transforming Growth Factor; TMC, N,N,N-trimethyl chitosan chloride; UCB-MSCs, Umbilical Cord Blood Mesenchymal Stromal Cells.

### 4.1 Ischemic and hypoxic environments

Tissues with limited oxygen supply, such as the heart and intervertebral discs, present severe survival barriers for transplanted MSCs. In the infarcted myocardium, hydrogels derived from Wharton’s Jelly ECM combined with MSCs of the same origin promoted engraftment and enhanced secretion of paracrine factors, which improved left ventricular function, reduced fibrosis, and stimulated neovascularization in rats (Tavajjohi et al., 2025). Similarly, bone marrow-derived MSCs encapsulated in RGD-modified alginate hydrogel showed improved retention and viability in infarcted rat hearts, leading to increased ejection fraction, reduced infarct size, and greater capillary density (Levit et al., 2013; Guo et al., 2020). Beyond structural support, gene-modified MSC therapies have also been investigated. Wu and colleagues (2017) demonstrated that gene-modified MSCs delivered within a small-molecule hydrogel significantly improved angiogenesis and reduced infarct size in a rat model of myocardial infarction (MI), underscoring the synergistic potential of combining MSCs with gene-activated hydrogels in ischemic repair. Comparable challenges exist in the intervertebral disc, where low oxygen and nutrient supply limit regenerative capacity. In a goat model of disc degeneration, hyaluronic acid-based hydrogels encapsulating MSCs restored disc height and stiffness and decreased local inflammation (Zhang et al., 2021). In spinal fusion, gelatin methacryloyl (GelMA) carriers were developed to co-deliver MSCs and bone BMP-2, promoting osteogenesis *in vitro* and bone bridging *in vivo* (Li et al., 2024). These approaches show that in hypoxic and avascular tissues, hydrogels provide a metabolic buffer that sustains MSC survival and activity while modulating inflammatory macrophage infiltration.

### 4.2 Neuroinhibitory niches and scar formation

In the central nervous system, regeneration is hindered by glial scar formation and a highly specialized immune environment dominated by microglia.

To address these barriers, Sun et al. (2025) engineered a thermosensitive hydrogel incorporating BMP7-loaded nanoparticles, which promoted neuronal differentiation of bone marrow MSCs, enhanced axonal regeneration, reduced glial scarring, and improved locomotor recovery in rats with spinal cord injury (SCI). In a complementary design, Papa et al. (2018) encapsulated MSCs in a hyaluronic acid hydrogel with controlled release of CCL2, which preserved spinal cord cytoarchitecture, reduced lesion volume, and recruited host cells. Importantly, this system induced macrophage polarization toward a regenerative phenotype, highlighting the immune-modulatory role of hydrogels in the central nervous system.

These findings indicate that hydrogel scaffolds in neuroregeneration must not only sustain MSC viability but also regulate microglia and infiltrating macrophages to counteract inhibitory scar formation.

### 4.3 Highly immune-active tissues

Tissues such as the skin and gut are highly exposed to inflammation and microbial stress, requiring MSC-hydrogel systems that prolong paracrine signaling and shape immune responses. In a diabetic mouse model, Huang et al. (2025) demonstrated that perinatal MSCs delivered via a hydrogel scaffold accelerated wound closure through the activation of the phosphoinositide 3-kinase/protein kinase B (PI3K/AKT) pathway. This resulted in the promotion of keratinocyte migration, angiogenesis, and proliferation. In atopic dermatitis, thiolated hyaluronic acid hydrogels formed *in situ* after injection, improving MSC engraftment and retention. In treated mice, epidermal hyperplasia and dermal inflammation were significantly reduced, with decreased immune cell infiltration (Lee et al., 2022). Similarly, in gastrointestinal injury, MSCs embedded in hydrogels containing heparan sulfate mimetics (HS-m) named RGTA^®^ showed to retain their immunoregulatory properties and reduced radiation-induced colonic inflammation, enhancing mucosal regeneration and restoring epithelial architecture (Moussa et al., 2019). Together, these studies show that in immune-rich tissues, hydrogels not only deliver MSCs but also provide a platform for reprogramming local macrophages and innate lymphoid cells, thereby facilitating resolution of inflammation.

### 4.4 Mechanically demanding and avascular tissues

Regeneration of cartilage, osteochondral interface, and bone is particularly difficult due to lack of vasculature, high mechanical stress, and poor intrinsic healing. In rabbits, Seol et al. (2013) reported that thermosensitive poloxamer 407 hydrogels carrying autologous MSCs filled full-thickness cartilage defects and promoted hyaline-like tissue formation with smooth surface morphology and improved histological scores. Hu and colleagues (2024) engineered a nanozyme-functionalized bilayer hydrogel conceptualized not only to promote osteochondral regeneration, but also to modulate the inflammatory microenvironment associate to tissue damage. The upper cartilage-inductive layer was composed of methacrylated gelatin (GelMA) incorporating kartogenin-loaded nanoparticles to stimulate chondrogenesis. The lower layer consisted of oxidized hyaluronic acid (OHA) and adipic acid dihydrazide (ADH) crosslinked hydrogel, embedded with manganese dioxide (MnO_2_)-based nanozymes to scavenge reactive oxygen species (ROS) and reduce local inflammation. This construct significantly enhanced cartilage and subchondral bone repair in rabbits.

Several studies have specifically explored gene-activated MSC scaffolds for osteochondral regeneration. Wang et al. (2010) implanted poly (lactic-co-glycolic acid) (PLGA) sponges filled with fibrin gel, bone marrow-derived MSCs, and DNA complexes into rabbit cartilage defects, achieving near-complete filling and formation of hyaline-like tissue. Chen et al. (2011) designed bilayered scaffolds with spatially controlled gene delivery, enabling simultaneous regeneration of articular cartilage and subchondral bone *in vivo*. Building on this, Li et al. (2013) fabricated PLGA scaffolds containing MSCs and plasmid DNA encoding transforming growth factor beta-1 (TGF-β1), which markedly enhanced cartilage repair quality and ECM deposition. In larger animal models, umbilical cord-derived MSCs embedded in hyaluronic acid hydrogels promoted cartilage repair in minipigs (Ha et al., 2015), while self-assembling peptide hydrogels delivering TGF-β1 induced MSC chondrogenesis and ECM deposition in equine cartilage defects (Kopesky et al., 2014).

Bone regeneration also benefits from gene-activated approaches. Loozen and colleagues (2019) demonstrated that BMP-2 gene delivery in both MSC-laden and even cell-free constructs enhanced osteogenesis in rodent models, showing that sustained morphogen release can substitute for continuous cellular input. Building on this principle, Lin et al. (2017) used projection stereolithography to fabricate BMP-2 gene-activated scaffolds with high spatial precision, achieving robust bone tissue formation. Similarly, Sun et al. (2020) applied an injectable BMP-2 gene-activated scaffold to successfully repair cranial bone defects in mice, highlighting the translational relevance of minimally invasive delivery systems. Beyond gene activation, clinically oriented MSC–hydrogel formulations have been developed to meet regulatory and translational requirements. Vivas and colleagues (2020) designed a good manufacturing practices (GMP)-compliant osteogenic preparation consisting of bone marrow-derived MSCs embedded in a fibrin matrix enriched with micronized bone particles (0.25–1 μm). When implanted subcutaneously in immunodeficient mice, the construct showed no signs of toxicity or adverse immune response, while supporting early osteogenesis and vascularization. Importantly, this work emphasized safety, scalability, and compliance with good manufacturing practices, underlining the feasibility of advancing MSC-hydrogel therapies toward clinical application. Complementing these findings, Black et al. (2020) created a hybrid scaffold that combined a bovine ECM-derived hydrogel with a melt-electro-written polycaprolactone (PCL) framework. In a bovine critical-sized bone defect model, this construct promoted not only extensive new bone formation but also vascular ingrowth and stable integration with host tissue.

In these mechanically demanding tissues, hydrogel systems not only provide structural integration but also coordinate MSC crosstalk with osteoclasts, osteoblasts, and osteal macrophages, underscoring the role of osteo-immunity in skeletal regeneration.

### 4.5 Immune-sensitive and specialized tissues

Certain tissues present unique regenerative challenges, requiring repair strategies that minimize local immune activation or support complex graft integration. In a preclinical rabbit model of vocal fold scarring, Bartlett et al. (2016) evaluated the therapeutic efficacy and safety of bone marrow-derived MSCs delivered alone or in combination with a hyaluronic acid-based hydrogel (HyStem-VF). The study highlighted that the injection of MSCs alone improved viscoelastic recovery and ECM remodeling, while MSC-hydrogel combinations unexpectedly triggered inflammation and impaired repair. This underscores that in specialized tissues, biomaterial selection must be carefully tuned to avoid counterproductive immune responses.

In pancreatic transplantation, Borg et al. (2016) developed polyethylene glycol (PEG)–heparin cryogels to co-deliver pancreatic islets and MSCs. Implanted subcutaneously in mice, this system preserved insulin secretion and prolonged graft survival, with MSCs providing paracrine and immunomodulatory support.

Gene-activated constructs have also been applied to skin appendages. Kolakshyapati and collaborators (2017) reported that MSCs seeded in gene-activated matrices regenerated sweat gland-like structures *in vivo*, suggesting potential for adnexal tissue repair in burn injuries.

### 4.6 Safety and translational considerations

Clinical translation of hydrogel-MSC systems demands not only therapeutic efficacy but also rigorous demonstration of safety, biocompatibility, and compliance with regulatory standards. Accordingly, Canceill et al. (2023) reported that platelet lysate–based fibrin hydrogels encapsulating MSCs were biocompatible when implanted in immunocompetent rats, showing no acute inflammation, toxicity, or ectopic tissue formation. Platelet-rich formulations, already rich in growth factors and anti-inflammatory mediators, are well-known to enhance MSC activity, serving as valuable xeno-free components that sustain cell viability and function, as well as support tissue repair and immune modulation in a physiologically relevant manner (Barbon et al., 2018; Stocco et al., 2019b). The preclinical data collected by Canceill et al. (2023) exemplify how clinically oriented matrices can serve as advanced therapy medicinal products (ATMPs), bridging the gap from preclinical testing to human trials.

## 5 Hydrogel-based MSC therapies: clinical research

**FIGURE 3:**
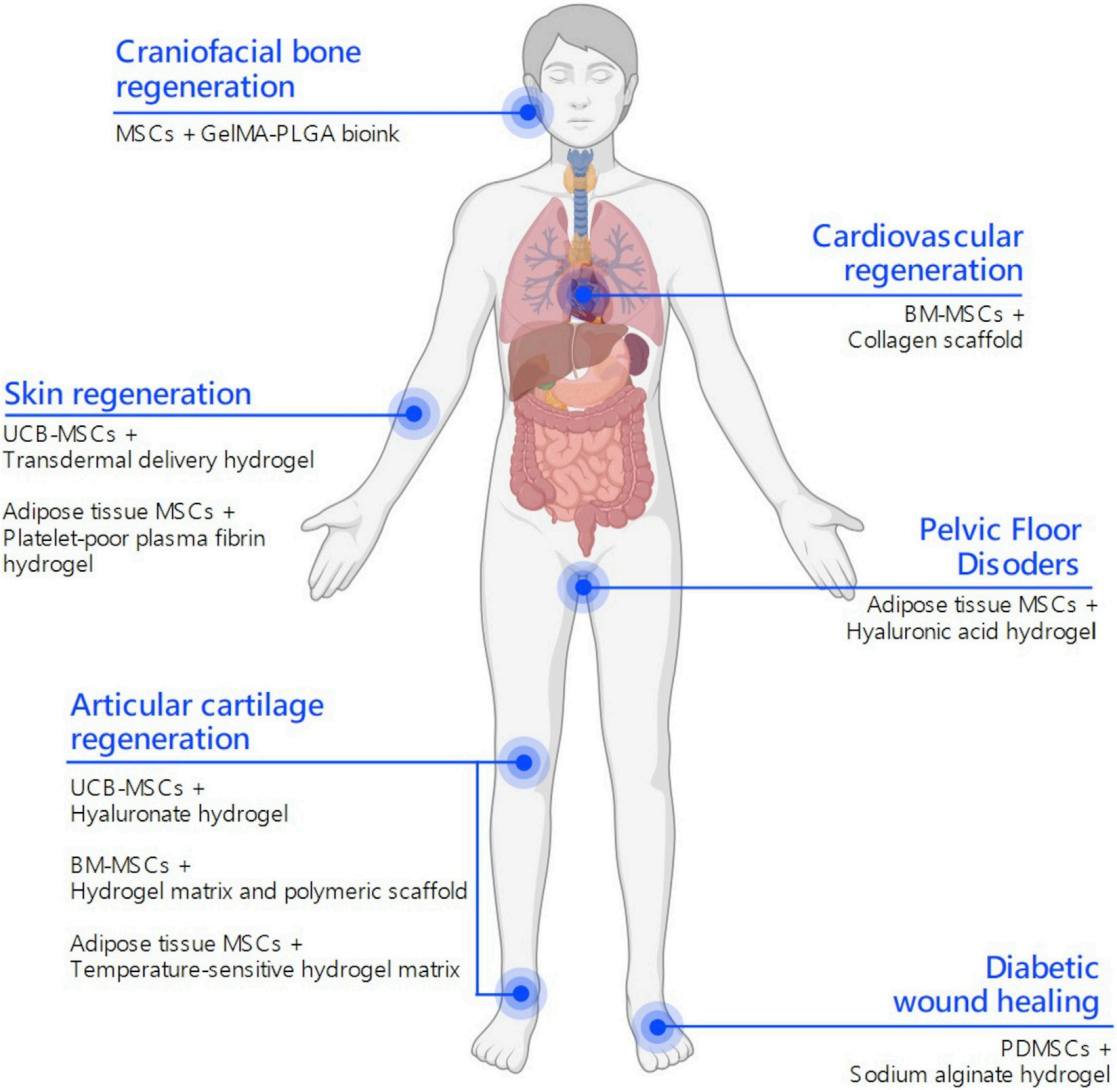
Clinical applications of MSC-laden hydrogels. Schematic representation of published and ongoing clinical trials on hydrogel-assisted mesenchymal stromal cell (MSCs) delivery to stimulate tissue regeneration. Depending on the anatomical site, distinct MSCs and hydrogel formulations are employed to address specific regenerative challenges. This integrative map highlights how hydrogel composition and MSC source can be strategically matched to different tissue microenvironments to improve regenerative outcomes. This figure was created using BioRender.com.

The clinical translation of MSC-based therapies has progressed significantly over the past decade, with numerous trials exploring their potential across various disease contexts. Nevertheless, when the integration of MSCs with hydrogel-based delivery systems is considered, clinical research appears to be in its early stages. Only a limited number of trials have investigated this combinatorial approach in human patients, with encouraging preliminary findings (Table 2). The following sections summarize representative clinical applications of hydrogel-assisted MSC delivery for cardiovascular, dermatological, articular, and chronic wound regeneration (Figure 3).

**TABLE 2:** Overview of MSC laden-hydrogel clinical research.

First author, year	Clinical target	Type of MSCs	Type of hydrogel	No. of patients	Surgery	Main clinical outcomes
Park et al. (2017)	Knee osteoarthritis	UCB-MSCs	Hyaluronate hydrogel	18	Intra-articular injection	- Cartilage regeneration - Pain relief - Joint function maintained over 7 years
Zeng et al. (2017)	Diabetic foot ulcer	PDMSCs	Sodium alginate hydrogel	1	-	- Accelerated wound healing - Re-epithelialization - Reduced inflammation
Fan et al. (2020b)	Cesarean section skin scars	UCB-MSCs	Transdermal delivery hydrogel	58	C-section (follow-up for scar management)	- Reduced scar thickness and erythema - Improved pliability
He et al. (2020)	Chronic ischemic heart disease	BM-MSCs	Collagen scaffold	114	CABG with MSC scaffold injection	- Improved myocardial perfusion - Greater LVEF - Increased 6-min walk distance

BM-MSCs, Bone Marrow Mesenchymal Stromal Cells; CABG, Coronary Artery Bypass Grafting; LVEF, left ventricular ejection fraction; MSCs, Mesenchymal Stromal Cells; PDMSCs, placenta-derived Mesenchymal Stromal Cells; UCB-MSCs, Umbilical Cord Blood Mesenchymal Stromal Cells.

### 5.1 Cardiovascular regeneration

The clinical translation of regenerative therapies for ischemic heart disease has taken into consideration the integration of cell-based interventions with biomaterial scaffolds to enhance therapeutic efficacy. As already mentioned, this combinatorial approach aims to address the limitations of poor cell retention, survival, and functional integration frequently observed with cell delivery alone. Within this framework, He et al. (2020) conducted a multicenter, randomized, double-blind, placebo-controlled clinical trial involving 114 patients with chronic ischemic heart disease. The intervention group received intramyocardial implantation of MSCs combined with a bioengineered collagen scaffold during coronary artery bypass graft surgery, while the control group received the surgery alone. Over a 12-month follow-up, patients treated with MSCs and scaffold showed significantly improved myocardial perfusion, greater left ventricular ejection fraction (LVEF), and increased 6-min walk distance compared to controls. Importantly, the procedure was well tolerated, with no increase in adverse events.

This trial offers compelling evidence that combining cell therapy with biomaterial scaffolds enhances functional outcomes in ischemic heart disease.

### 5.2 Skin and scar regeneration

Post-surgical scarring, especially hypertrophic or keloid scars following cesarean sections, presents significant aesthetic and functional concerns. Traditional treatments have limited success in promoting satisfying dermal regeneration. MSCs, with their anti-inflammatory and remodeling capabilities, offer a novel approach to improving scar quality.

Recently, Fan et al. (2020b) conducted a randomized, double-blind, placebo-controlled clinical trial to assess the efficacy of umbilical cord-derived MSCs for improving cesarean section scar outcomes. Fifty-eight women were enrolled and randomly assigned to receive intradermal injections of MSCs or placebo along the surgical incision site. The stem cells were delivered in a transdermal hydrogel formulation designed to enhance local retention and therapeutic activity.

At 6 months post-treatment, scar assessments were performed using clinical examination, histological analysis, and the Vancouver Scar Scale (VSS). Results indicated modest improvements in scar appearance among MSC-treated patients, including reduced erythema, improved pliability, decreased scar thickness, and enhanced collagen remodeling. However, these differences did not reach statistical significance compared to the placebo group. Importantly, the treatment was well tolerated, with no adverse events or immune-related reactions reported.

These results suggest that UC-MSCs may be a safe and effective treatment to modulate fibroproliferative responses in human skin wounds. Beyond cosmetic benefits, this approach may reduce functional limitations and discomfort associated with scarring. However, larger trials are needed to standardize dosing and administration strategies.

### 5.3 Articular cartilage repair

Degenerative diseases affecting articular cartilage, such as osteoarthritis, are among the most common sources of chronic pain and functional impairment worldwide. Conventional treatments (pharmacologic management, physiotherapy, and surgical interventions) primarily aim to alleviate symptoms but fail in restoring cartilage integrity or halting disease progression. In this context, the integration of MSCs with biocompatible scaffolds, particularly hydrogels, represents a promising regenerative strategy. As part of this growing interest in cartilage regeneration therapies, Park and colleagues (2017) conducted an open-label, single-arm clinical trial in 18 patients with knee osteoarthritis. The treatment consisted of a composite product containing allogeneic umbilical cord blood-derived MSCs mixed with hyaluronic acid hydrogel, injected intra-articularly. Clinical and arthroscopic assessments over 7 years showed sustained improvement in pain and mobility, with imaging revealing the development of hyaline-like cartilage and joint space preservation.

Together, these outcomes emphasize the long-term regenerative potential of MSC-hydrogel therapies in osteoarthritic conditions.

### 5.4 Diabetic wound healing

Chronic non-healing wounds such as diabetic foot ulcers are a major complication of diabetes, often leading to infection and amputation. Innovative therapies that promote re-epithelialization and vascularization are urgently needed. MSCs have appear to be promising therapeutic candidates due to their regenerative and immunomodulatory effects, particularly when delivered in a hydrogel vehicle.

In this scenario, Zeng et al. (2017) described a single-case application of placenta-derived MSCs embedded in a hydrogel matrix applied topically to a chronic diabetic foot ulcer. The wound was previously unresponsive to standard therapies. Over a 3-week period, the ulcer showed accelerated granulation tissue development, reduced local inflammation, and almost complete re-epithelialization. No complications were observed.

Despite being a case report, the study demonstrates the translational feasibility of topical MSC-hydrogel therapies. The favorable outcome supports further investigation through controlled trials. Numerous polymeric biomaterials are used recently for diabetic wound dressing including hydrogels, collagen-based scaffolds, whose efficacy can be further improved by loading therapeutic molecules, growth factors, and anti-microbial agents that could accelerate the process of wound closure by triggering collagen deposition and vascularization. However, advanced research in this field is essential to enhance the efficacy of polymeric wound dressings for efficient diabetic wound treatment (Sathyaraj et al., 2023). The selection of placenta-derived MSCs suggests a potential for enhanced immunomodulatory action and broader clinical applicability due to their allogeneic compatibility.

### 5.5 Ongoing clinical trials on MSC-laden hydrogels

Although the number of published clinical trials investigating hydrogel-assisted MSC therapies remains limited, other ongoing studies registered on the databases *ClinicalTrials.gov*, *EudraCT* (European Union Drug Regulating Authorities Clinical Trials) and *EU Clinical Trials Register* underscore the growing translational advancement in this field. These trials explore different hydrogel platforms, cellular sources, and delivery strategies, reflecting the adaptability of MSC-laden hydrogels across a range of regenerative approaches (Table 3).

**TABLE 3:** Ongoing clinical trials on MSC laden-hydrogels for tissue regeneration.

Identification number (study start year)	Clinical target	MSC type	Hydrogel type	Delivery route	Phase and status
NCT03113747 (2013)	2° or 3° degree burn wounds	Allogeneic MSCs from adipose tissue	Platelet-poor plasma fibrin hydrogel	Localized topical application to burn wound sites	Phase I/II Recruitment status “Unknown” *since last update in April 2017*
EudraCT 2021-002331-34 (2022)	Anal sphincter defect and chronic FI	Adipose tissue MSCs	Hyaluronic acid hydrogel	Intralesional injection	Phase IIb Status: Ongoing
NCT06028763 (2023)	Focal cartilage lesions in the ankle joint	Autologous MSCs from adipose tissue	Temperature-sensitive hydrogel matrix	Intra-articular injection of the cell-hydrogel composite into the knee joint	Phase I, Interventional Status: Recruiting
EudraCT 2024-512977-28-01 (2024)	Knee femoral cartilage lesions	Autologous BM-MSCs	Hydrogel matrix and polymeric scaffold	Local application within the knee joint	Phase I Status: Not yet recruiting
NCT06533150 (2024)	Craniofacial bone defects	Potential combination with MSCs	GelMA-PLGA bioink	Local implantation during craniofacial reconstructive surgery	Phase I, Interventional Status: Active, not yet recruiting

BM-MSCs, Bone Marrow MSCs; Mesenchymal Stromal Cells; FI, fecal incontinence; GelMA, Gelatin methacryloyl (GelMA); MSCs, Mesenchymal Stromal Cells; PLGA, poly (lactic-co-glycolic acid).

In the context of autologous regenerative therapies, the multicenter, exploratory trial NCT03113747 entitled “Allogeneic ADSCs and Platelet-Poor Plasma Fibrin Hydrogel to Treat the Patients With Burn Wounds” investigates the safety and preliminary efficacy of a tissue-engineered construct combining allogeneic adipose tissue MSCs embedded in a platelet-poor plasma (PPP)-derived fibrin hydrogel. This study targets patients with deep second- or third-degree burns covering 10%–50% of their body surface. The composite graft is applied directly to wounds within 24 h post-injury to evaluate its potential to accelerate healing and reduce scarring. Specifically, the PPP-derived hydrogel aims to improve local MSC retention and bioactivity, thereby enhancing tissue healing responses. Currently, the study status is listed as “Unknown” due to the absence of updates since April 2017. This suggests possible interruption or prolonged inactivity, underscoring the criticisms often encountered in cell-based product development and clinical translation (Voronin, 2025).

Despite these challenges, further clinical research efforts are being pursued to validate and optimize regenerative therapies based on MSC-laden hydrogels. For example, the EudraCT 2021-002331-34 trial is a multicenter, randomized, double-blind Phase IIb study which evaluates the safety and efficacy of allogeneic adipose-derived MSCs embedded in a hyaluronic acid hydrogel for the treatment of structural fecal incontinence (FI). Patients with a confirmed anal sphincter defect and chronic FI were administered with the investigated MSC-hydrogel product via intralesional injection, comparing 2 cell doses (60 million vs. 120 million adipose tissue-derived MSCs) suspended in a hyaluronic acid matrix. This trial exemplifies the growing clinical interest in MSC-laden hydrogels as a novel treatment strategy for sphincter repair, combining regenerative potential of stem cells with their localized, minimally invasive delivery. Its outcomes may offer valuable insights into the clinical applicability of cell-based biomaterial therapies in pelvic floor disorders (FIMABIS, 2025).

The recent trial NCT06028763 titled “Development of Biomedical Technology for the Treatment of Ankle Cartilage Using Injectable Biocomposite Hydrogel” focuses on adipose tissue-derived MSCs embedded within a customized hydrogel matrix and administered locally for the treatment of ankle cartilage focal injuries. These lesions are particularly difficult to treat due to the avascular nature and limited intrinsic healing capacity of cartilage tissue. The trial addresses these challenges by delivering MSCs in a minimally invasive, injectable hydrogel that solidifies at body temperature, enabling mechanical support, targeted delivery and long-term cellular retention within the ankle joint (Center of National Scientific Center of Traumatology and Orthopedics, 2025).

The clinical challenge of articular cartilage regeneration is also being addressed by a recently registered Phase I clinical trial (EudraCT 2024-512977-28-01), designed to evaluate the safety and feasibility of a novel advanced therapy medicinal product (ATMP). This investigational approach combines autologous bone marrow-derived MSCs with a biocompatible hydrogel matrix and a polymeric scaffold for the treatment of focal femoral condyle cartilage lesions in the knee. The trial aims to assess not only the local tolerability of the composite implant, but also the operational feasibility of its GMP-compliant manufacturing and surgical application. Depending on its specific outcomes, this study may represent a key step toward the clinical translation of MSC-laden hydrogel systems in orthopedic regenerative medicine (Lamina Therapeutics, 2025).

Finally, the trial NCT06533150 titled “Functionalized Bioink Delivering Biomolecules for the Treatment of Craniofacial Diseases (DART-CRAFT)” is developing an advanced Gelatin methacryloyl (GelMA) and poly (lactic-co-glycolic acid) (PLGA)-based bioink for potential MSC encapsulation and 3D bioprinting applications in craniofacial bone repair procedures. The bioink is applied intraoperatively during reconstructive surgery, where it serves as a moldable and *in situ* solidifying scaffold, tailored to support bone regeneration by enhancing local tissue integration and possibly incorporating MSCs. This innovative approach merges 3D bioprinting, controlled release technology, and biocompatible materials to address complex craniofacial defects with high precision. By enabling localized therapeutic delivery of growth factors or cells and structural support, the DART-CRAFT platform highlights the future of personalized regenerative strategies in maxillofacial surgery (Fondazione Policlinico Universitario Agostino Gemelli IRCCS, 2025).

Collectively, these ongoing trials demonstrate the continued evolution and clinical interest in hydrogel-based MSC delivery systems, highlighting their potential to enhance therapeutic efficacy, standardization, and applicability across multiple regenerative medicine applications.

## 6 Potential research gaps

Despite significant advancements in the preclinical and clinical application of MSC-laden hydrogels for tissue regeneration, several research gaps hinder their full clinical translation. One major challenge is the lack of standardized protocols for hydrogel composition, including optimal mechanical properties, degradation rates, and bioactive molecule incorporation, which vary depending on the target tissue (Burdick and Mauck, 2011). Additionally, while MSC-hydrogel systems have shown promise in preclinical studies, there is limited long-term data on cell survival, engraftment, and functional integration in human clinical trials (Galipeau and Sensébé, 2018). Another critical gap is the insufficient understanding of the paracrine mechanisms by which MSCs exert their therapeutic effects within hydrogels. While MSCs are known to secrete regenerative factors, the precise signaling pathways and their modulation by the hydrogel microenvironment remain unclear (Caplan and Correa, 2011). Furthermore, immune responses to implanted MSC-laden hydrogels, including potential foreign body reactions or unintended immunomodulatory effects, require further investigation (Andrzejewska et al., 2019).

Scalability and manufacturing consistency also pose challenges, as variations in MSC sources (e.g., autologous vs. allogeneic) and hydrogel fabrication techniques can impact therapeutic outcomes (Murphy et al., 2020). Finally, regulatory and ethical considerations surrounding MSC-based therapies necessitate clearer guidelines to facilitate clinical adoption (Sipp et al., 2018). Addressing these gaps through interdisciplinary research will be crucial for advancing MSC-laden hydrogels toward widespread clinical use in tissue regeneration.

## 7 Future clinical research focus

The clinical application of MSC-laden hydrogels holds immense potential for tissue regeneration, yet future research must address key challenges to optimize therapeutic efficacy and translation. A critical focus will be the development of smart hydrogels with tunable biomechanical and biochemical properties that can dynamically respond to the regenerative microenvironment (Daly et al., 2021). Such advanced biomaterials should enhance MSC retention, survival, and controlled differentiation while minimizing immune rejection (Zhu et al., 2022). Importantly, MSC-based therapies alone have shown to often fail to achieve long-term and uncomplicated wound healing across different tissues, largely due to the transient survival of transplanted cells and the short-lived duration of their paracrine signaling. Embedding MSCs within hydrogels directly addresses this limitation, as these systems can provide a supportive matrix that prolongs MSC bioactivity, sustains the release of anti-inflammatory and trophic factors, and better synchronizes cellular functions with the extended phases of tissue repair and remodeling.

Building on this rationale, another priority is the standardization of MSC sources and hydrogel formulations for specific clinical indications, such as osteoarthritis, chronic wounds, and myocardial infarction. Large-scale, randomized controlled trials (RCTs) are needed to validate the safety and long-term functional outcomes of MSC-hydrogel therapies compared to conventional treatments (Wei et al., 2023). Additionally, integrating technologies like 3D bioprinting and gene editing could enable patient-specific constructs with spatially controlled MSC delivery (Gungor-Ozkerim et al., 2018).

Understanding the immune-modulatory mechanisms of MSCs within hydrogels will also be essential, particularly in allogeneic applications, to prevent adverse responses and harness their anti-inflammatory potential (Levy et al., 2020). Finally, regulatory frameworks must evolve to accommodate the unique challenges of combination products (cells + biomaterials), ensuring reproducibility and scalability for commercialization (Mendicino et al., 2022). By addressing these priorities, future clinical research can unlock the full regenerative potential of MSC-laden hydrogels.
